# Pressured to be proud? Investigating the link between perceived norms and intergroup attitudes in members of disadvantaged minority groups

**DOI:** 10.1111/bjso.12874

**Published:** 2025-03-04

**Authors:** Juliane Degner, Joelle‐Cathrin Flöther, Iniobong Essien

**Affiliations:** ^1^ Department of Social Psychology University of Hamburg Hamburg Germany; ^2^ Leuphana Universität Lüneburg Lüneburg Germany

**Keywords:** group attitudes in disadvantaged groups, ingroup preference, outgroup preference, social norms, System Justification Theory

## Abstract

System Justification Theory (SJT) proposes that members of disadvantaged groups perceive norms to express ingroup positivity. Adherence to these norms is assumed to result in open expressions of ingroup preferences on self‐report measures while being unrelated to ingroup preferences assessed with indirect measures. We tested these assumptions with members of three disadvantaged groups: participants who identified as Gay or Lesbian (*n* = 196), as Black or African American (*n* = 202), or who reported higher weight (*n* = 208). We tested hypotheses on perceived norms and group attitudes at the individual level as well as at the social group level. While results at the group level suggest that differences in group attitudes between different disadvantaged groups are indeed related to differences in social norm perceptions between these groups, no consistent interrelations between norm perceptions and group attitudes were found at the individual level. We discuss the implications of these results, questioning SJTs basic postulate of group attitudes as manifestations of system justification processes in members of disadvantaged groups. We further argue that future research in this domain requires improved conceptual clarity in current theorizing, along with improved methodological operationalizations.

## INTRODUCTION

System Justification Theory (SJT; Jost & Banaji, 1994) postulates that individuals are inherently motivated to accept, justify, and defend societal structures, even when these are disadvantageous to themselves or their groups. One presumed manifestation of these motivations is group attitudes within disadvantaged groups: According to SJT, members of disadvantaged groups harbour negative ingroup attitudes and preferences for outgroups but conceal those attitudes due to perceived social norms to express ingroup preference (Jost et al., 2004). The current research investigates this proposed role of social norms for group attitudes among members of disadvantaged groups.

### Group attitudes among members of disadvantaged groups

Belonging to a disadvantaged group poses substantial psychological challenges for navigating one's social identity and group attitudes. According to Social Identity Theory (SIT, Tajfel & Turner, 1979), individuals seek a positive social identity, achieved by favouring their ingroup and employing diverse strategies of motivated group comparisons to enhance the ingroup's status and evaluation (i.e. social creativity). However, when confronted with enduring disadvantage, low status, or stigmatization, the application of such strategies may not yield positive evaluations of the ingroup. Consequently, SIT proposed that members of disadvantaged groups either strive to distance themselves from their ingroups to safeguard individual self‐esteem (i.e. upward individual mobility) or pursue group‐level strategies aimed at improving ingroup status via system change (i.e. collective action; see de Lemus & Stroebe, 2015 for an overview). System Justification Theory (SJT; Jost, 2019, 2020; Jost et al., 2004; Jost & Banaji, 1994) was introduced as an extension of the social identity approach, presenting an alternative perspective on how disadvantaged groups respond to low ingroup status. SJT postulates that members of disadvantaged groups endorse and justify the lower status associated with their ingroup rather than seeking to avoid or change it.

Specifically, SJT proposes that individuals possess inherent motivations to support and defend societal arrangements, even when they are disadvantageous to them or their ingroup (Jost et al., 2004). These motivations cause individuals to internalize societal beliefs and attitudes, and exhibit behaviours that legitimize the status quo of the existing social system. For members of disadvantaged groups, this implies attributing the lower status of their ingroup to internal characteristics and forming negative attitudes towards the ingroup and preference for advantaged outgroups, termed “*outgroup favoritism*” or “*outgroup preference*” (H6, Jost, 2020, p. 115), resulting from an “*internalization of inferiority*” (Jost, 2020, p. 110).

While the current research focuses on postulates of SJT, it is important to note that there are alternative theoretical frameworks regarding outgroup preferences in disadvantaged groups, such as the Social Identity Model of System Attitudes (SIMSA; Owuamalam et al., 2016, 2018, 2019a, 2019b; Rubin et al., 2023). SIMSA suggests that outgroup preferences among members of disadvantaged groups may simply reflect a passive acknowledgment of the status quo rather than an active endorsement or defence of it. Additionally, outgroup preferences may serve various strategies to maintain or enhance ingroup status. Thus, according to SIMSA, explaining outgroup preferences among disadvantaged group members does not require postulating a separate system justification motive as a driving factor.

The aim of the current work is not to take a stance for or against either theory. Instead, we address a central auxiliary assumption within SJT that has not yet received empirical attention: When presuming internalized inferiority among individuals from disadvantaged groups, SJT additionally postulates that they refrain from openly expressing outgroup preferences due to social norms encouraging the display of ingroup preferences instead.^1^ According to SJT, these norms may be especially strong among members of disadvantaged groups that have faced discrimination and prejudice over generations (e.g. Black Americans; Jost, 2020). SJT suggests that hidden outgroup preferences nevertheless remain accessible and can be detected when using indirect, unobtrusive and/or implicit measures, which are presumed to be less influenced by self‐presentation or social desirability concerns (formulated in H6′ in Jost et al., 2004; H7 in Jost, 2020; cf. Owuamalam et al., 2019a). SJT thus postulates a dissociation of outcomes of different measures, with ingroup preference being observed on direct self‐report measures and outgroup preference being observed on indirect unobtrusive measures.

SJT offers one further rationale for this hypothesized dissociation, proposing that internalized stigma may operate at unconscious levels: Members of disadvantaged groups may not be consciously aware of their internalized negative attitudes towards their ingroup and thus unable to express them. These unconscious attitudes may nevertheless affect thoughts and behaviours and be detectable by implicit measures, whereas self‐report measures are thought to reflect the effects of compliance with ingroup positivity norms. These assumptions highlight both conceptual ambiguity regarding the processes underlying internalized inferiority, and methodological ambiguity regarding implicit measures and their interpretability, as we outline in the Discussion section.

Extensive research has examined group attitudes among members of disadvantaged groups, but findings only partially support SJT's predictions. For example, early research conducted with Black and African Americans using evaluative Implicit Association Tests (IATs; Greenwald et al., 1998) has documented outgroup preferences in some studies (e.g. Ashburn‐Nardo et al., 2003; Nosek et al., 2002) and ingroup preference in others (e.g. Livingston, 2002). A meta‐analysis by Essien et al. (2021) documented a high level of heterogeneity of group attitudes within and between different disadvantaged groups: Some groups consistently displayed ingroup preferences on both direct and indirect measures (e.g. Gay and Lesbian participants), some showed inconsistent patterns of ingroup and outgroup preferences on direct and indirect measures (e.g. Black and African Americans), and other groups consistently displayed outgroup preferences on direct and indirect measures (e.g. overweight participants). Such patterns of results appear inconsistent with SJT predictions: Given that *all* of the included groups were negatively evaluated by non‐group members, internalization of inferiority as postulated in SJT should result in (various levels) of outgroup preference in all of these groups.

Although not explicitly stated in System Justification Theory (SJT), the heterogeneity in group attitudes within and across different disadvantaged groups may be partly attributed to heterogeneity in group norms regarding group attitudes. Therefore, the current research aims to investigate whether group attitudes among members of disadvantaged groups are indeed related to perceptions of social norms.

### Group evaluation norms in disadvantaged groups

To our knowledge, previous research has not systematically investigated whether members of disadvantaged groups actually perceive normative pressure towards expressing ingroup preferences. To support this assumption, SJT writing (e.g. Jost, 2020; Jost et al., 2004) has primarily referenced research conducted by Miller and Ratner (Miller, 1999; Miller & Ratner, 1998; Ratner & Miller, 2001). However, their work examined perceived norms of *self*‐interest but not norms of (in)*group*‐interest, as suggested by SJT.

As further evidence for the operation of group evaluation norms, Jost et al. (2004) referenced a minimal group experiment by Scheepers et al. (2002). This experiment found that participants assigned to a lower‐status minimal group viewed an ingroup member who claimed that status differences were legitimate as less valuable than a neutral ingroup member and were more inclined to elect the neutral member as a leader. These findings may indeed be interpreted as an effect of devaluation of ingroup members who deviated from an ingroup preference norm. However, the devaluation effect in the lower‐status condition was small and non‐significant and the study was underpowered to test such small effect size. It thus appears inappropriate to refer to these findings as support for the conclusion that “*(p)rescriptive norms to avoid “identification with the oppressor” and the “Uncle Tom” syndrome can be intense*.” (Jost et al., 2004, p. 893).

More recent findings by Iacoviello and Spears (2022) suggest that ingroup preferences in minimal groups may indeed be influenced by inferred ingroup norms promoting ingroup favouritism. In this experiment, preference for a minimal ingroup was greater when people inferred or imagined approval of their fellow ingroup members than when they imagined approval of an external entity. However, since this research did not implement any group status manipulation, it leaves open whether members of disadvantaged or lower‐status groups might also perceive and conform to such ingroup positivity norms.

Taken together, there is a lack of empirical evidence for the idea that members of disadvantaged groups perceive strong ingroup norms to display ingroup preferences, as most research on normative effects has focused on members of majorities and/or advantaged groups (cf. Rubin et al., 2023). The only available evidence is based on minimal group studies, which either report null effects (Scheepers et al., 2002) or do not manipulate ingroup status (Iacoviello & Spears, 2022).

Given the lack of systematic research on this topic, it appears crucial to investigate perceptions of social norms of group attitudes among members of real‐world disadvantaged groups. However, we first need to address some conceptual ambiguities in SJT regarding the types of social norms, their reference groups, and types of norm conformity before introducing our own research.

#### Identifying and locating normative pressure

Theorizing on social norms presents a range of theoretical distinctions, some of which are relevant to the postulates of System Justification Theory (SJT). First, norms are related to specific reference groups, which also affect their relative impact on people's attitudes and behaviours (e.g. Smith & Louis, 2009). For example, the postulated normative pressure to exhibit ingroup preference may be considered an *ingroup* norm (i.e. communicated within the ingroup) or a more generic or superordinate *societal* norm (i.e. communicated by society). The relative impact of ingroup norms and societal norms may differ: Prior research indicates that ingroup norms are more effective in guiding thought and behaviour than societal norms, especially in individuals who identify strongly with the ingroup (e.g. Liu et al., 2019; White et al., 2009). This has been explained by self‐categorization and social identification processes that transform the group from an external entity to a fundamental aspect of the self, increasing its relevance for individual decisions (Spears, 2021). While SJT does not explicitly designate the postulated group attitude norm to a reference group, we interpret SJT as prioritizing the ingroup over society (“The expression of ingroup rather than outgroup favouritism might well be encouraged by social norms, especially among members of devaluated groups”, Jost, 2020, p. 118).

Second, norms are typically differentiated into *descriptive* versus *injunctive* norms (Cialdini et al., 1990). Descriptive norms refer to individual perception of others' behaviour or attitudes, thus representations of what people *tend to do* and what is common or typical. Injunctive norms (also termed *prescriptive* norms) refer to standards, guides, or expectations of desired, correct, or appropriate behaviour, thus representations of what people *ought to do* (e.g. Gavrilets, 2020). Both types of norms can play important roles in shaping attitudes and behaviour, but descriptive norms tend to have a somewhat stronger influence on behaviour than injunctive norms (e.g. White et al., 2009; see Anderson & Dunning, 2014; Legros & Cislaghi, 2020 for reviews). SJT does not specify whether the suggested normative pressure for expressing ingroup preferences arises from descriptive or injunctive norms. Generally, SJT suggests “injunctification”, in that system justification motives encourage individuals to treat societal descriptive norms of “what is” as standard for societal injunctive norms of “what ought to be” (e.g. Jost et al., 2015; Kay et al., 2009; cf. Deutchman et al., 2025). From this perspective, one might anticipate little distinction between descriptive and injunctive societal norms. However, SJT does not explicitly address whether injunctification also applies to ingroup norms.

A third relevant distinction relates to how social norms affect individual behaviour: *private* conformity, where people personally adopt and use a norm as a personal standard, and *public* conformity, where people only outwardly comply with normative pressure without truly accepting the norm (e.g. Sowden et al., 2018). While SJT does not make explicit references to these terms, its conceptualization of *internalized inferiority* suggests private conformity with the ingroup's societal status. Furthermore, when postulating that members of disadvantaged groups openly express ingroup preference while secretly harbour outgroup preferences, SJT seems to suggest an effect of mere public conformity without private acceptance of ingroup preferences.

Conceptualizations of private versus public conformity somewhat align with SJT's predictions regarding differentiations between different attitude measures. Public compliance is expected to affect open self‐report measures if these are susceptible to self‐presentation motivation and social desirability concerns. In contrast, private conformity is expected to affect responses in non‐reactive, indirect, or unobtrusive measures if these are less susceptible to self‐presentation motivation and social desirability concerns and thus reflect privately held attitudes. In the Discussion section of this paper, we thoroughly reevaluate these assumptions regarding implicit measurement characteristics.

### The current research

The major aim of the current research was to provide empirical evidence on ingroup preference norm perceptions among members of disadvantaged groups and investigate their relations to group attitudes. We collected data in a cross‐sectional survey design in three disadvantaged groups in the United States: people who identified as Gay or Lesbian, people who identified as Black or African American, and people who identified as being Overweight. These groups were selected based on meta‐analytic results (Essien et al., 2021) to represent the high variability of group preferences and stigmatization.

We operationalized perceived ingroup preference norms both as ingroup norms as well as societal norms. For ingroup norms, we further differentiated between descriptive norms and injunctive norms. Additionally, we implemented direct and indirect measures of group preferences and ingroup evaluations.

Data collections were pre‐registered as three independent studies at the Open Science Framework (https://osf.io/jrt7v/), where all materials, methods, data, and analysis scripts are publicly available. The study protocol was approved by the ethics committee of the Department of Psychology at the University of Hamburg.

We pre‐registered three hypotheses: First, to the extent that group attitude measures represent internalized stigma, we should observe group attitudes based on the group's relative level of stigmatization. Based on prior meta‐analytic findings (Essien et al., 2021), we expected significant effects of ingroup preference in the sample of Gay and Lesbian participants (H1a) as well as in the sample of Black and African American participants (H1b), and significant effects of outgroup preference in the sample of participants with higher weight (H1c). Based on previously documented correspondence between direct and indirect measures at the group level (Essien et al., 2021), we did not pre‐register any hypotheses regarding differences between measures. Note that according to SJT (Jost et al., 2004), however, participants would be expected to exhibit outgroup preferences on indirect measures and ingroup preferences on direct measures.

Second, we hypothesized that deliberate expressions of ingroup preferences in the direct measures should be positively correlated with perceived injunctive group evaluation norms (H2).

Third, ingroup preferences on indirect measures should be uncorrelated with perceived group evaluation norms (H3).

We had pre‐registered standard individual‐based analytical approaches for each study, separately examining within‐group variance in group attitudes and norm perceptions and their interrelations for each group. We extended this approach by additional group‐based analyses to explore the relationship between group‐level differences, which may be equally informative for testing SJT. Further pre‐registered exploratory analyses are reported in Appendix S1.

## METHOD

### Sample size determination

For all three samples, we had pre‐registered to provide sufficient test power (1 − *β* = .80 at *α* < .05) to test for *r* = .20 as the smallest effect size of interest for the correlation between ingroup preference measures and norm perception measures, thus requiring valid data from 191 participants per sample. Participants were recruited using Prolific (https://www.prolific.co/). Studies 1 and 2 were initially commenced by 250 Prolific users each; Study 3 was commenced by 255 users. Of these, *n*_1_ = 204, *n*_2_ = 212, and *n*_3_ = 214 participants completed the data collection and provided informed consent for data storage and analyses (see Procedures). One pre‐registered inclusion criterion was related to social group membership. We further applied pre‐screening criteria based on country of residence (USA, excluding the data of *n*_1_ = 8, *n*_2_ = 10, *n*_3_ = 8 participants with non‐US citizenship), first language (English), and no participation in any other study of our lab. Note that for all three studies, we had originally pre‐registered to exclude data of participants who failed an attention check more than once (*n*_1_ = 2, *n*_2_ = 15, *n*_3_ = 19). Because the exclusion of these participants did not alter results in any meaningful way, we decided to deviate from this exclusion criterion and included data of these participants to increase the test power of our results. There were no further exclusions of participants, but for 13 participants, the IAT data were classified as invalid (see Measures).

### Participants

The current analyses rely on valid data of 606 participants from the following three samples.

#### Gay and Lesbian participants

We obtained valid data of *N* = 196 persons who self‐identified on Prolific as Gay or Lesbian (of which 90 self‐categorized as female, 98 as male, and eight as diverse or non‐binary). The majority of participants, *n* = 144 (73.5%), identified as White, 19 (10%) as Asian, 15 (8%) as Black, eight (4%) as Hispanic or Latinx, and 10 (2%) as other. Participants' age ranged from 18 to 70 years (*Md* = 28, *M* = 30.9, *SD* = 11.75).

#### Black and African American participants

We obtained valid data of *N* = 202 persons who self‐identified on Prolific as Black or African American (108 self‐categorized as female, 92 as male, two as diverse or non‐binary). Participants' ages ranged from 18 to 75 years (*Md* = 27, *M* = 31.0, *SD* = 11.75).

#### Higher weight participants

We obtained valid data of *N* = 208 participants who were registered on Prolific with a BMI > 35^2^ and self‐identified in our survey as overweight (130 self‐categorized as female, 74 as male, 4 as diverse or non‐binary, excluding 31 participants who did not self‐categorize as overweight). The majority of participants, *n* = 183 (88%), identified as White, nine (4%) as Black, eight (4%) as Hispanic or Latinx, five (2%) as Asian, and three (1.5%) as other. Participants' age ranged from 18 to 66 years (*Md* = 34, *M* = 35.6, *SD* = 11.42). Participants' body mass index calculated from self‐reported weight and height ranged from 24.55 to 62.85 (*M* = 40.03, *SD* = 8.29). Thirty‐eight participants (18.3%) self‐categorized as slightly overweight, 93 (44.7%) as moderately overweight, and 77 (37.0%) as extremely overweight.

### Measures

#### Implicit association tests

We created three IATs adapted for use in Qualtrics (http://iatgen.org/; Carpenter et al., 2019). In each IAT, participants categorized 20 attribute words as *Good* versus *Bad* and 20 target images as belonging to one of the target categories. In Sample 1, the target categories were *Gay* versus *Straight*, each represented by 10 images of same‐gender (five female, five male) and 10 images of different‐gender couples in romantic, yet non‐sexual poses (e.g. holding hands) from a commercial photo stock. In Sample 2, the target categories were *Black people* versus *White people*, each represented by 10 portrait pictures selected from the Chicago Face Database (Ma et al., 2015). In Sample 3, the target categories were *Overweight persons* versus *Normal‐weight persons*, each represented by 10 morphs of facial portraits created from three individuals from Google image searches.

For the attribute categories, we used 10 words with positive meanings (*adore*, *happy*, *hope*, *joyous*, *lovely*, *luck*, *magnificent*, *pleasure*, *spectacular*, *triumph*) and 10 words with negative meaning (*angry*, *awful*, *despise*, *disaster*, *grief*, *hate*, *horrible*, *nasty*, *tragic*, *war*).

The IAT consisted of seven blocks, with a random assignment of which target–attribute pairings came first. We used the analysis tool provided by iatgen to calculate an IAT *D*‐score (the D600 algorithm, Greenwald et al., 2003). *D*‐scores above zero indicate faster responses when the ingroup targets and good attributes versus outgroup targets and bad attributes shared a response key compared to when the key assignment was the opposite. *D*‐scores are typically interpreted as an indicator of relative ingroup preference. We excluded IAT data of 13 participants (*n*_1_ = 3, *n*_2_ = 5, *n*_3_ = 5) with ≥10% of trials with response times ≤300 ms (excessive speed criterion; see Greenwald et al., 2003). All IATs had satisfying reliability indices with *α*_1_ = .905, *α*_2_ = .864, *α*_3_ = .862.

#### Self‐reported group evaluations

##### Intergroup preference

For reasons of comparability with previous research (e.g. Essien et al., 2021), we employed a one‐item group preference measure with a 7‐point scale, ranging from outgroup preference (i.e. “I strongly prefer Straight/White/normal‐weight people to Gay/Black/Overweight people”) via no preference (i.e. “I like Gay/Black/Overweight people and Straight/White/normal‐weight people equally”) to ingroup preference (i.e. “I strongly prefer Gay/Black/Overweight people to Straight/White/normal‐weight people”).

##### Ingroup evaluation

As a measure of non‐comparative ingroup identity evaluation, we adapted five items from Luhtanen and Crocker (1992), assessing positive and negative ingroup evaluations (e.g. “I am proud to be Gay/Black/Overweight.”, “I regret being Gay/Black/Overweight.”). We reverse‐scored negative attitude items such that higher mean scale values indicate more positive ingroup evaluations. The scale had satisfying reliabilities, *α*_1_ = .841, *α*_2_ = .810, *α*_3_ = .870.

##### Norm measures

We created measures of evaluation norms by adapting the formulations of the five ingroup evaluation items to reflect perceived descriptive ingroup norms (e.g. “Most Gay/Black/Overweight people are proud to be Gay/Black/Overweight.”, *α*_1_ = .739, *α*_2_ = .769, *α*_3_ = .849), injunctive ingroup norms (e.g. “Most Gay/Black/Overweight people expect other Gay/Black/Overweight people to be proud to be Gay/Black/Overweight.”, *α*_1_ = .670, *α*_2_ = .706, *α*_3_ = .812), and injunctive societal norms (e.g. “Society expects Gay/Black/Overweight people to be proud to be Gay/Black/Overweight.”, *α*_1_ = .762, *α*_2_ = .824, *α*_3_ = .757).

##### Exploratory measures

The study included additional exploratory measures of ingroup identity centrality (2 items), system‐justifying beliefs (8 items), group status perception (2 items) and political orientation (1 item). We describe these measures and report their exploratory analyses in Appendix S1 (section S.3).

### Procedure

Data collection was conducted in June and July 2020 using *Qualtrics* (www.qualtrics.com). Upon recruitment on *Prolific*, participants were informed that the study investigated social group attitudes and their relations to group identification. The study started with a welcome page that probed initial consent for participation and data storage and contained an attention check. Participants who failed the attention check were asked to re‐read the instructions.

Participants then completed the IAT, followed by the one‐item group preference measure. Next, participants reported demographic information (age, gender, nationality), including self‐categorizations with regard to the social identity (sexual orientation, racialized group membership, and weight status, respectively). Participants whose self‐categorizations did not match sample inclusion criteria were forwarded to the end of the survey, and their data collected up to this point was excluded from analyses. The remaining survey started with the two ingroup identification centrality items (see section S.3 in Appendix S1) and five ingroup evaluation items. Each item appeared together with the three norm‐related variants assessing perceived injunctive ingroup norms, perceived descriptive ingroup norms, and perceived injunctive societal norms. The survey then continued with the exploratory measures (see Appendix S1). For all measures reported here, responses were collected on 7‐point Likert‐type scales ranging from 1 (*strongly disagree*) to 7 (*strongly agree*). After survey completion, participants were fully debriefed about the registered hypotheses and confirmed (or withdrew) their initial consent for data storage and analyses.

## RESULTS

Our pre‐registered analyses plan only specified analyses at the individual level. However, we additionally performed group‐level analyses to provide a more comprehensive test of SJTs postulates. We first report group means and group differences in the preference measures (H1) as well as norm measures. We then report hypothesis tests with regard to interrelations between the group evaluation measures and their relationship to participants' norm perceptions (H2 and H3).

### Intergroup preference measures

#### IAT scores

The three samples showed substantial differences in their *D*‐scores, *F*(2, 588) = 97.810, *p* < .001, $\eta_{p}^{2}$ = .250, 90% CI = [0.200, 0.295], see Table 1. Gay and Lesbian participants yielded a significant positive average *D*‐score with a moderate effect size, *t*(191) = 7.146, *p* < .001, *d* = 0.516, 95% CI = [0.365, 0.666] indicating ingroup preference; Black and African Americans average *D*‐score was not significantly different from zero, *t*(194) = −1.767, *p* = .079, *d* = −0.127, 95% CI = [−0.267, 0.015], indicating no group preference; and higher weight participants yielded a significant negative average *D*‐score with a large effect size, *t*(203) = −12.176, *p* < .001, *d* = −0.853, 95% CI = [−1.008, −0.689], indicating outgroup preference.

**TABLE 1:** Group evaluation measures.

Sample	IAT *D*‐score^a^			Intergroup preference^b^			Ingroup evaluation^b^		
	*M*	*SD*	*d*	*M*	*SD*	*d*	*M*	*SD*	*d*
Gay or Lesbian	0.248	0.481	0.516	5.367	1.227	1.114	5.767	1.018	1.737
Black or African American	−0.052	0.412	−0.127	4.960	1.229	0.781	5.900	0.968	1.962
Higher weight	−0.380	0.446	−0.853	3.707	0.991	–0.296	2.046	1.035	−1.888

*Note*: Cohen's *d* stem from *t*‐tests against zero for the IAT and against the scale midpoints of the self‐report measures.

^a^ Positive values indicate ingroup preference.

^b^ Values above 4 (midpoint of the scale) indicate ingroup preference or positive ingroup identity evaluation.

#### Self‐reported intergroup preference

The three samples also exhibited substantial differences in their self‐reported group preferences, *F*(2, 603) = 115.013, *p* < .001, $\eta_{p}^{2}$ = .276, 90% CI = [0.227, 0.891]; see Table 1. We tested participants' scores against the scale midpoint and observed that Gay and Lesbian participants expressed a significant large effect of ingroup preference, *t*(195) = 15.602, *p* < .001, *d* = 1.114, 95% CI = [0.935, 1.292]. Black and African Americans also expressed a significant large effect of ingroup preference, *t*(194) = 11.105, *p* < .001, *d* = 0.781, 95% CI = [0.623, 0.938]. Higher weight participants expressed a significant small‐to‐medium effect of outgroup preference, *t*(203) = −4.270, *p* < .001, *d* = −0.296, 95% CI = [−0.435, −0.157].

#### Self‐reported ingroup evaluation

The three samples showed substantial differences in the ingroup evaluation scale, *F*(2, 603) = 966.874, *p* < .001, $\eta_{p}^{2}$ = .762, 90% CI = [0.738, 0.782]; see Table 1. Comparisons against the scale midpoint revealed that both Gay and Lesbian participants and Black and African American participants expressed substantial positive ingroup evaluations, *t*(195) = 24.313, *p* < .001, *d* = 1.737, 95% CI = [1.514, 1.958], and *t*(201) = 27.891, *p* < .001, *d* = 1.962, 95% CI = [1.725, 2.198], respectively. Higher weight participants, on the other hand, expressed significant negative ingroup evaluations, *t*(203) = −27.236, *p* < .001, *d* = −1.888, 95% CI = [−2.115, −1.661], with a large effect size.

In summary, the results of the group evaluation measures supported our hypotheses regarding group differences (Essien et al., 2021) but were less consistent with predictions from SJT (Jost et al., 2004): Only the results of Black and African American participants somewhat confirmed SJT expectations of positive ingroup evaluations on direct measures and no group preference on indirect measures. Gay and Lesbian participants exhibited ingroup preference and positive ingroup evaluation on all measures, and higher weight participants exhibited outgroup preference and negative ingroup evaluation on all measures.

### Group evaluation norms

#### Descriptive ingroup norms

There were significant group differences with regard to the perceived descriptive ingroup norm, *F*(2, 603) = 812.751, *p* < .001, $\eta_{p}^{2}$ = .729, 90% CI = [0.702, 0.752], see Table 2. We tested participants' scores against the scale midpoint and observed that Black and African American participants reported the most positive descriptive ingroup evaluation norm, *t*(201) = 25.695, *p* < .001, *d* = 1.808, 95% CI = [1.583, 2.031], followed by Gay and Lesbian participants, *t*(195) = 18.516, *p* < .001, *d* = 1.323, 95% CI = [1.130, 1.514], with large effect sizes for both samples. Higher weight participants, on the other hand, expressed significant negative descriptive ingroup evaluation norms, *t*(203) = −26.113, *p* < .001, *d* = −1.811, 95% CI = [−2.031, −1.589], with a large effect size.

**TABLE 2:** Group evaluation norm measures.

	Descriptive ingroup norm			Injunctive ingroup norm			Injunctive societal norm		
	*M*	*SD*	*d*	*M*	*SD*	*d*	*M*	*SD*	*d*
Gay or Lesbian	5.143	0.864	1.323	5.375	0.772	1.779	3.709	1.097	−0.265
Black or African American	5.598	0.884	1.808	5.889	0.759	2.488	4.179	1.368	0.131
Higher weight	2.314	0.931	−1.811	2.668	1.034	−1.289	2.268	1.038	−1.669

*Note*: Effect size estimates Cohen's *d* stem from *t*‐tests against the scale midpoints (4). Values above 4 indicate agreement with positive group evaluation norms, values below 4 indicate disagreement, respectively.

#### Injunctive ingroup norms

We observed similar group differences for perceptions of injunctive ingroup norms, *F*(2, 603) = 817.707, *p* < .001, $\eta_{p}^{2}$ = .731, 90% CI = [0.703, 0.753], see Table 2. Black and African American participants expressed the most positive injunctive ingroup norms, *t*(201) = 35.368, *p* < .001, *d* = 2.488, 95% CI = [2.208, 2.767], followed by Gay and Lesbian participants, *t*(195) = 24.907, *p* < .001, *d* = 1.779, 95% CI = [1.553, 2.003], with very large effect sizes. Higher weight participants expressed significantly negative injunctive ingroup evaluation norms, *t*(203) = −18.588, *p* < .001, *d* = −1.289, 95% CI = [−1.472, −1.104]. For all three samples, injunctive norms of positive ingroup evaluation were perceived as stronger than descriptive norms, *t*s >3.877, *p*s < .001, *d*s > .277, and both types of ingroup norms were positively correlated *r*_1_ = .482, *r*_2_ = .710, and *r*_3_ = .767, *p*s < .001, respectively. This suggests that the measures of descriptive and injunctive norm perceptions may largely reflect the same or highly interrelated underlying construct(s).

#### Injunctive societal norms

We also observed significant group differences with regard to participants' perceptions of injunctive societal norms of group evaluations, *F*(2, 603) = 147.313, *p* < .001, $\eta_{p}^{2}$ = .328, 90% CI = [0.278, 0.372], see Table 2. Whereas Black and African American participants exhibited a small but non‐significant effect of positive injunctive societal norms of group evaluation, *t*(201) = 1.861, *p* = .064, *d* = 0.131, 95% CI = [−0.008, 0.269], Gay and Lesbian participants expressed some disagreement with the idea of societal norms for positive group evaluation, *t*(195) = −3.712, *p* < .001, *d* = −.265, 95% CI = [−0.407, −0.122], and higher weight participants expressed strong disagreement, *t*(203) = −24.073, *p* < .001, *d* = −1.669, 95% CI = [−1.879, −1.458].

To summarize, participants in the Gay and Lesbian sample as well as in the Black and African American sample reported descriptive and injunctive norms to express positive ingroup evaluations but disagreed with the idea of positive injunctive societal norms. Participants of the higher weight sample, on the other hand, reported negative descriptive and injunctive ingroup norms and negative injunctive societal norms.

### Interrelations of group evaluations and evaluative norm perceptions

**FIGURE 1:**
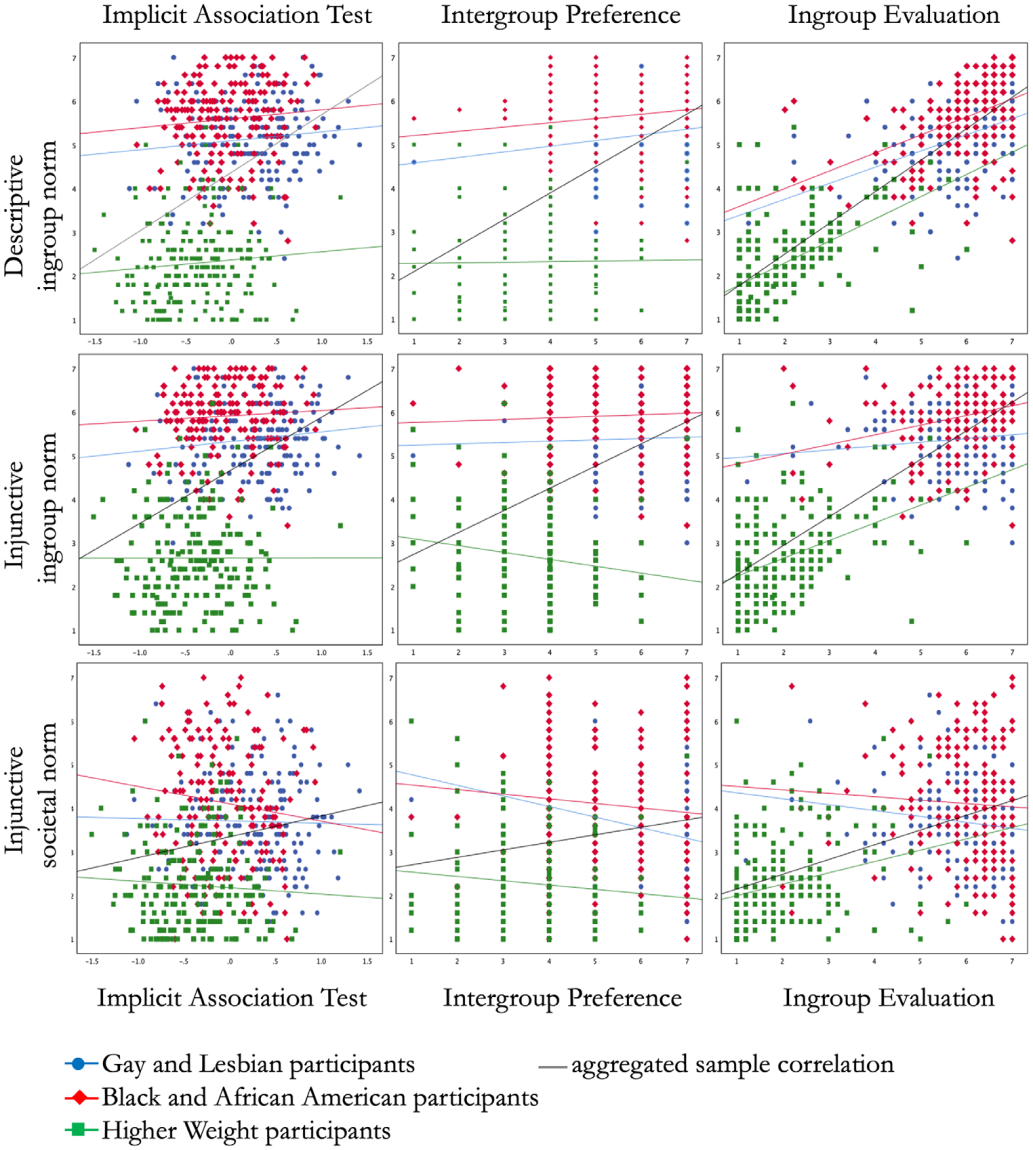
Scatterplots depicting interrelations of group evaluation measure and norm perceptions.

For hypotheses tests of interrelations between group evaluations and norm perceptions, we had originally pre‐registered separate within‐group correlations for each sample. We additionally report correlations for the combined samples along with partial correlations controlling for subsample, which allow consideration of potential between‐group differences^3^ (see Figure 1). In Appendix S1, we additionally report hierarchical multiple regression analyses for each group evaluation measure, testing for moderation effects of the relationships between group evaluation measures and norm perceptions by type of group, respectively (section S.1 in Appendix S1) and further multiple regression analyses simultaneously including all three norm measures as predictors of each group evaluation measure to explore independent effects of these norms (section S.2 in Appendix S1).

#### IAT *D*‐scores

As can be seen in Table 3, IAT scores were significantly related to all norm measures at the combined‐sample level, but partial correlations controlling for subsample were non‐significant. Thus, while the three samples substantially differed in their norm perceptions, which in turn were related to group differences in group preferences, within‐sample differences in *D*‐scores were unrelated to perceptions of evaluation norms (see Figure 1, left panels). The multiple regression analyses (S.1.1 in Appendix S1) confirmed these sample differences and further demonstrated that the relations between *D*‐scores and norm perceptions were not moderated by sample. Further multiple regressions testing for the independent versus interactive effects of ingroup and societal norms (S.2.1 in Appendix S1) revealed independent predictive values of all norm measures but also indicated pronounced between‐group differences in all three norm variables.

**TABLE 3:** Correlation coefficients *r* (*p*‐values) for the relation between group attitude measures and norm perception measures—with partial correlations controlling for the sample.

	Evaluative norm perceptions		
	Descriptive ingroup norm	Injunctive ingroup norm	Injunctive societal norm
IAT scores			
Overall (bivariate)	.392 (.000)	.372 (.000)	.169 (.000)
Overall (partial)	.078 (.057)	.054 (.189)	−.055 (.182)
Gay or Lesbian	.112 (.121)	.138 (.056)	−.023 (.747)
Black or African American	.082 (.253)	.072 (.320)	−.117 (.104)
Higher weight	.091 (.195)	.001 (.990)	−.064 (.360)
Intergroup preference			
Overall (bivariate)	.474 (.000)	.412 (.000)	.166 (.000)
Overall (partial)	.207 (.000)	.116 (.005)	−.048 (.242)
Gay or Lesbian	.183 (.010)	.048 (.506)	−.272 (.000)
Black or African American	.162 (.021)	.088 (.213)	−.085 (.229)
Higher weight	.014 (.842)	−.151 (.029)	−.094 (.176)
Ingroup evaluation			
Overall (bivariate)	.867 (.000)	.814 (.000)	.501 (.000)
Overall (partial)	.732 (.000)	.632 (.000)	.315 (.001)
Gay or Lesbian	.435 (.000)	.115 (.110)	−.126 (.079)
Black or African American	.471 (.000)	.303 (.000)	−.040 (.573)
Higher weight	.564 (.000)	.407 (.000)	.260 (.000)

To summarize, IAT effects were positively related to both descriptive and injunctive ingroup norms at the combined‐sample level but not at the individual within‐sample level. They were positively related to injunctive societal norms at the group level but tended to be negatively related to societal norms at the individual within‐group level, trending towards a Simpson's Paradox (Simpson, 1951). Thus, it appears that indirectly measured group preferences tended to positively align with both descriptive and injunctive ingroup norms of positive group evaluation but tended to negatively align with societal norms.

#### Self‐reported intergroup preference

At the combined‐sample level, we observed significant positive correlations between the one‐item intergroup preference measure and the descriptive and injunctive ingroup norm perceptions, but a much smaller positive relationship to injunctive societal norms (see Table 3). Partial correlation coefficients were substantially reduced for the ingroup norm measures and insignificant for the societal norm measure, indicating that these relationships were largely explained by between‐group differences in group preference and norm perceptions (see Figure 1, central panels).

Moderation analyses (S.1.2 in Appendix S1) confirmed moderation effects by sample: Within‐sample variance in descriptive ingroup norm perceptions was significantly related to the open expression of ingroup preference in the sample of Gay and Lesbian participants as well as in the sample of Black and African American participants, indicating that ingroup preference was positively related to perceptions of a descriptive norm of ingroup positivity. No significant correlation was observed in the sample of higher weight participants. The pattern for injunctive ingroup norms was different: There were no significant correlations within the Gay and Lesbian sample and the Black and African American sample, and there was a significant negative correlation in the higher weight sample indicating that participants tended to express more outgroup preference the more they perceived an injunctive ingroup norm towards ingroup preference. Finally, there was a significant moderation by subsample for injunctive societal norms: While all within‐sample correlation coefficients were negative, this relationship was only significant in the Gay and Lesbian sample, indicating that Gay and Lesbian participants expressed lower levels of ingroup preference the more they perceived injunctive societal norms to evaluate the ingroup positively.

Additional multiple regressions testing the independent effects of each norm measure on self‐reported intergroup preferences (see S.2.2 in Appendix S1) revealed independent predictive values of the descriptive ingroup norm and of the injunctive societal norm, but no independent effect of injunctive ingroup norms.

To summarize, at the combined‐sample level, self‐reported intergroup preferences were positively related to ingroup norms as well as societal norms, whereas at the individual within‐sample level, intergroup preferences were only inconsistently related to norm perceptions.

#### Self‐reported ingroup evaluations

There were significant large positive correlations between the ingroup identification evaluation scale and all norm measures, which were somewhat reduced in partial correlations controlling for subsamples. Note that these correlations may be artificially inflated because the norm measures employed the identical item formulation from the group evaluation scale adapted to measure norm perceptions.

The relationship between positive ingroup evaluations and descriptive ingroup norms was not significantly moderated by sample (section S.2.3 in Appendix S1): We observed large, significant correlations in all three samples. Thus, the expression of positive ingroup evaluations was strongly related to perceptions of a descriptive ingroup norm of group evaluation at the individual and the group level.

The relationship between injunctive ingroup norm perceptions and positive ingroup evaluations was partly moderated by subsample (section S.2.3 in Appendix S1): Bivariate correlations calculated separately for each sample revealed a non‐significant positive correlation in the Gay and Lesbian sample, a significant medium‐sized positive correlation in the Black and African American sample, and a significant large positive correlation in the higher weight sample (see Table 3). Finally, correlations with injunctive societal norms again showed a pattern of a Simpson's Paradox (Simpson, 1951): While there were positive correlations at the combined‐sample level, the within‐sample correlation differed for each subsample (see Table 3). We observed non‐significant correlations in both the Gay and Lesbian sample and the Black and African American sample, and a significant positive correlation in the higher weight sample.

A multiple regression analysis including all three norm measures as simultaneous predictors of the open expression of positive ingroup evaluations yielded independent predictive values for all three norm measures (see section S.2.3 in Appendix S1).

To summarize, at the combined‐sample level, positive ingroup evaluations were strongly positively related to ingroup norms but were inconsistently related to within‐sample variances of societal norms.

## DISCUSSION

The primary aim of this research was to examine group attitudes and evaluation norms among members of disadvantaged groups and the interrelations of these variables, marking the first study directly addressing these associations within real disadvantaged groups, as posited by SJT (Jost, 2020).

As we had expected, group attitudes among members of disadvantaged groups varied between groups and measures: Gay and Lesbian participants exhibited ingroup preferences on direct and indirect measures; Black and African American participants exhibited ingroup preferences on direct measures but no group preference on the indirect measure; higher weight participants exhibited outgroup preferences on both measures. These findings are inconsistent with SJT, which posits that disadvantaged group members generally exhibit outgroup preferences on indirect measures due to internalized inferiority and exhibit ingroup preferences on direct measures due to conformity to ingroup positivity norms (Jost, 2020). The observed group differences, however, replicate findings from a recent meta‐analysis (Essien et al., 2021, see also Degner et al., 2021).

Analyses of norm perceptions revealed that some disadvantaged groups reported indeed positive ingroup evaluation norms as postulated by SJT. Specifically, Gay and Lesbian participants and Black and African American participants reported positive ingroup evaluation norms with little distinction between descriptive and injunctive ingroup norms. Higher‐weight participants, in contrast, reported negative descriptive and injunctive ingroup norms. We further observed that *ingroup norm* perceptions were significantly related to group evaluations, but these effects were mainly driven by between‐group variation and less by within‐group variation. These results are somewhat in line with research with members of advantaged groups documenting that ingroup norms affect the expression of group attitudes and ingroup preference behaviours (e.g. Crandall et al., 2002; Jetten et al., 1996, 1997).

Findings with regard to societal norm perceptions were more mixed: We observed a certain degree of norm conformity at the group level, indicating that the group reporting the most negative societal evaluation norm (i.e. higher weight people) also exhibited outgroup preference and negative ingroup evaluation, whereas groups reporting less negative societal norms tended to exhibit ingroup preferences and positive ingroup evaluation. These relationships were partly reversed at the individual within‐group level, especially among Gay and Lesbian and Black and African American participants: Participants perceiving more negative societal norms exhibited more ingroup preferences and positive ingroup evaluation. Again, correlational patterns were highly similar for the direct and indirect group evaluation measures.

One may conclude that the group‐level results support SJT postulates: Only groups with positive ingroup evaluation norms openly expressed ingroup preferences and positive ingroup evaluation, whereas those with negative ingroup evaluation norms expressed outgroup preferences and negative ingroup evaluation. Furthermore, group attitudes appeared more closely related to ingroup norms than to societal norms. However, contrary to SJT hypotheses, we did not observe pronounced measurement dissociations, as these relationships were similar for direct and indirect measures of group attitudes.

It is important to highlight that the main function of the auxiliary assumption concerning normative pressure to express ingroup positivity in SJT was to explain observed discrepancies between self‐reported and indirectly measured group attitudes (Hypotheses H6′ in Jost et al., 2004; H7 in Jost, 2019). If we interpret the IAT‐scores as indicators of internalized attitudes (but see discussion below), we should have thus observed a dissociation between IAT and self‐reported attitudes to be explained by public conformity without private compliance to ingroup evaluation norms. Specifically, Gay and Lesbian participants – who reported strong ingroup positivity norms – should have expressed ingroup preference in the self‐report measure but outgroup preference in the indirect measure. However, they exhibited ingroup preference and positive ingroup evaluations across *all* measures. Similar effects were observed in Black and African American participants, who also did not exhibit outgroup preference or ingroup derogation. The only group demonstrating outgroup preferences were higher weight participants, but they exhibited this tendency across all measures. Importantly, this group was not characterized by higher system justification beliefs or more conservative ideologies than the other groups (see Appendix S1), which would have been expected based on the interindividual difference perspective of SJT. Our main conclusion here is that there was no pattern of hidden outgroup preference or ingroup derogation based on ingroup norm conformity in any of the three groups.

### Theoretical relevance

Based on our findings and in line with other meta‐analytic evidence (Essien et al., 2021), we suggest that SJT may strike assumptions of outgroup preferences as a *general* manifestation of system justification tendencies among members of disadvantaged groups. While there certainly are groups and individual members of disadvantaged groups who exhibit outgroup preferences and ingroup derogation, these effects may be better explained by group‐specific and/or individual processes rather than stemming from an overarching system justification motive, as others have suggested before (e.g. Owuamalam, et al., 2016, 2019; Rubin et al., 2023). For example, our exploratory analyses (see Appendix S1) suggest that observed group‐level differences may be partly attributable to differences in ingroup centrality: Groups whose members reported positive ingroup evaluation norms and exhibited ingroup preferences (i.e. Gay and Lesbian participants) or no group preference (i.e. Black and African American participants) exhibited significantly higher ingroup centrality than higher weight participants who exhibited outgroup preference and negative group evaluation norms – a finding consistent with Social Identity theorizing. There may be further group‐specific and superordinate factors affecting ingroup attitudes and evaluation norms among disadvantaged group members (e.g. Owuamalam et al., 2019a, 2023; Rubin et al., 2023). Prior research has documented that group attitudes among members of disadvantaged groups closely align with the relative strength or severity of group stigmatization in a given society (Essien et al., 2021). Similarly, the observed group differences may be attributable to social reality constraints related to cultural representations of groups. For example, Charlesworth and Banaji (2019) demonstrated that societal biases towards sexual minorities and racial groups in the USA have declined over the past decade and are projected to continue declining. In contrast, weight bias has remained consistently high and is projected to persist. The authors suggest that such trends relate to different levels of public discourse which influence societal trends towards egalitarianism towards specific groups (Charlesworth & Banaji, 2019). Furthermore, they may impact perceptions of social consensus and societal constraints such as the normative acceptability of stigmatization (e.g. Crandall et al., 2002; Rubin et al., 2023), as well as differences in the perceived morality, legitimacy, and stability of group status differences (e.g. Jost, 2019; Oaten et al., 2022) or system dependency (Kay et al., 2009). Finally, characteristics of the specific stigma associated with disadvantaged groups (e.g. varying concealability, disruptiveness, or controllability of stigma; e.g. Pachankis et al., 2018), characteristics of the groups (e.g. their perceived entitativity, or permeability; Armenta et al., 2017; Rydell & McConnell, 2005), or differences in group members' stigma consciousness (e.g. Flöther & Degner, 2025; Pinel, 1999) may be responsible for group differences in attitudes. For example, one may speculate that high levels of perceived permeability and controllability might impact higher weight individuals' attitudes towards the ingroup as well as the formation and communication of evaluation norms compared to less permeable social groups where stigma is perceived as less controllable (e.g. sexual minorities and racial groups; see Armenta et al., 2017). The resulting low identity centrality inhibits the operation of any group‐enhancing strategies to create positive ingroup evaluations.

Our conclusions regarding SJT align with the main tenet of SIT (Tajfel & Turner, 1979) and SIMSA (Owuamalam et al., 2016, 2018; Rubin et al., 2023) in that explaining outgroup preferences among members of disadvantaged groups does not require postulating additional system justification motives. Instead, outgroup preferences may align with either self‐ or group interests. For example, the ingroup negativity and outgroup preference in higher weight participants may align with SIMSAs accuracy argument if we assume that social constraints of stigma are stronger for weight‐related stigmatization than for the stigmatization of sexual and racial minorities in the USA, given societal prioritization and control beliefs. Forming negative attitudes towards the ingroup may be a simple acknowledgment of the ingroup's low societal status. If body weight is believed to be under personal control, weight‐related stigma can be perceived as legitimate derogation of an individual's lack of control and discipline, legitimizing stigma (see for similar arguments Owuamalam et al., 2019a; Reicher, 2004; Rubin & Hewstone, 2004). Given an increasing focus on health and the obesity epidemic, there is less public debate and societal prioritization of weight stigma (Charlesworth & Banaji, 2019); and a lack of education regarding the *systemic* causes of overweight and limited individual controllability. Thus, stigma legitimizing controllability beliefs may not be challenged in public discourse.

Given that System Justification Theory aims to explain group attitudes among members of disadvantaged groups, future work should clarify the conditions and psychological mechanisms that lead to differences between groups. Greater theoretical clarity would not only aid in researching influencing variables but also enhance our understanding of the relationship between group attitudes and evaluation norms and help explain the heterogeneity in group attitudes within disadvantaged populations.

### Limitations

First, the correlational nature of our findings prohibits conclusions regarding directional causal relationships between the measured variables as postulated in SJT. While it is possible that the formation of group attitudes follows from conformity to group evaluation norms, a reversed causal pathway seems equally possible: Members of disadvantaged groups may anchor their perceptions of prevailing evaluative norms in their own group attitudes (e.g. Cadinu & Rothbart, 1996; Otten & Wentura, 2001). We can also construe a bidirectional causal relationship between these two factors. Alternatively, the relationships between group attitudes and group norms may be explained by third variables affecting both the level of relative group stigmatization in society as well as the formation of ingroup attitudes and evaluation norms in members of disadvantaged groups, including variations in the characteristics of groups, group stigma, and public discourse as outlined above.

Second, the inconsistencies between group‐level and individual‐level results may seem like a puzzling limitation to the interpretability and generalizability of the current results: Relationships between group attitudes and ingroup norms were mainly attributable to between‐group effects that are not replicated at the individual level within the three groups. However, we presume that this apparent inconsistency is mainly attributable to the reduced variances within the three samples. A closer look at Figure 1 shows that while group attitude measures differed substantially between groups and these differences covaried with group differences in ingroup norm perceptions, the variance of these measures within the samples was rather low—limiting the general interpretability of the correlational approach. This limited variance may have resulted from inadequate sampling if we presume, for example, that Prolific users who self‐categorize as members of these disadvantaged groups do not represent their ingroup populations well. However, we deem it possible and plausible that such limited variance is indeed a true characteristic of (some) disadvantaged groups whose members may highly agree with their norm perceptions as well as their group attitudes. Further research with a representative sampling approach is needed to estimate the true variance of these variables among members of disadvantaged groups.

### Outlook

Social norms have played an important role in research on intergroup attitudes since the establishment of this research domain (e.g. Spears, 2021), although they often ignore the specific perspectives of lower‐status and/or disadvantaged groups. We believe that it would be highly fruitful to enhance theorizing and research about the role of social norms in these groups, especially because they may face very different normative conflicts than other social groups (cf. Flöther & Degner, 2025). We consider it crucial to improve conceptual clarity, operational procedures, and methodology in this endeavour. Given the high impact and popularity of System Justification Theory, we highlight two interrelated aspects of the theory that we believe require improvement beyond the aforementioned theoretical implications: the theoretical conceptualization of internalized inferiority and the use of direct and indirect measures to assess various attitude mechanisms.

#### Conceptualizing internalized inferiority

Current formulations of SJT provide an ambiguous conceptualization of internalized inferiority. It is conceptualized as both a personally held belief and as an unconscious attitude that is “soundly rejected at an explicit level” (Jost, 2020, p. 119). The former matches conceptualizations of internalized stigma in the sociological and psychological literature on stigma as the acceptance or endorsement of negative stereotypes, prejudice, and attitudes, and their incorporations into one's self‐identity or self‐concept (e.g. Burke et al., 2019; Livingston & Boyd, 2010; Quinn & Earnshaw, 2011). The latter matches early theoretical conceptualizations of implicit attitudes as “introspectively unidentified traces of experience” (Greenwald & Banaji, 1995, p. 8; cf. Hahn & Goedderz, 2020). The conceptual ambiguity caused by these two different conceptualizations may have remained unnoticed because both were used for identical predictions with regard to measurement dissociations (Hypothesis 6′, Jost et al., 2004; Hypothesis 7, Jost, 2019): Outgroup preference is expected to be more likely observed on indirect measures than self‐reports because indirect measures are (a) either more likely to detect privately held beliefs unaffected by self‐presentation concerns or (b) more sensitive to detect the automatic activation of an introspectively unidentified or unconscious attitude. Similarly, outgroup preference is expected to be less likely to be observed in direct self‐report measures, because participants either (a) are aware of their outgroup preference but unwilling to openly express it or (b) are unaware that they harbour such attitudes. Thus, the same pattern of results is expected regardless of whether it arises from people being *unwilling* or *unable* to openly express ingroup derogation or outgroup preferences. However, when explaining underlying psychological mechanisms, such as the effects of social norms on the formation and expression of group attitudes in disadvantaged group members, it appears crucial to disambiguate and sharpen the definition of the concept.

In the literature on social norms, internalization is typically defined as (a) the personal endorsement and acceptance of a norm as valid, and (b) as behavioural alignment with that norm, which occurs independently of social rewards or sanctions (e.g. Gavrilets, 2020; Gavrilets & Richerson, 2017; Gross & Vostroknutov, 2022; Spears, 2021). SJT, however, primarily emphasizes the behavioural aspect, presuming that it occurs without requiring conscious personal endorsement. It is not entirely implausible that individuals may align their behaviours with personal standards without being aware of these standards, as people sometimes lack insight into the internal causes of their attitudes and behaviours (e.g. Nisbett & Wilson, 1977; Timmermans & Cleeremans, 2015; but see Gawronski & Corneille, 2025). However, we question the theoretical necessity of assuming the operation of unconscious norms in the formation and expression of group attitudes as postulated in SJT. Instead, we propose a more parsimonious definition of internalized stigma as the personal acceptance or endorsement of negative stereotypes, prejudices and attitudes, without making additional assumptions regarding individuals' awareness. This approach aligns with existing literature on internalized stigma (e.g. Burke et al., 2019; Livingston & Boyd, 2010; Quinn & Earnshaw, 2011).

Even with this more parsimonious definition, a key question remains: When ingroup and societal norms conflict, why and under which conditions would members of disadvantaged groups internalize societal norms (resulting in internalized stigma) rather than ingroup norms (resulting in ingroup pride)? This postulate rests on SJT's assumption that system justification motives can take precedence over group and ego motives, particularly when their personal and/or group identities are low in salience or strength. However, our results do not support this premise: Heavyweight individuals, who demonstrated the greatest alignment between their attitudes and societal perceptions of their ingroup and the lowest levels of identity centrality, did not exhibit higher system justification beliefs than participants belonging to sexual or racial minorities, who explicitly distanced themselves and their ingroup from negative societal norms and exhibited higher identity centrality (see Appendix S1). Moreover, since the literature widely indicates that ingroup norms generally hold more influence on behaviour than societal norms (e.g. Ellemers et al., 2002; Spears, 2021; White et al., 2009), we see no reason to believe that this would differ for members of disadvantaged groups.

#### Direct and indirect measures

The theoretical ambiguities regarding the conceptualization of internalized inferiority as unconscious attitudes are deeply intertwined with methodological issues, specifically with SJT's assumption that implicit and explicit measures differ in their sensitivity to automatic, associative, and/or unconscious processes versus intentional, deliberate, and/or conscious processes. Our results do not support assumptions of such conceptual or operational dissociations (see also Essien et al., 2021).

While dualisms regarding types of attitudes (conscious vs. unconscious) and measures (explicit vs. implicit, controlled vs. automatic) were prominent and prevalent in social cognition theorizing and research at the time that SJT was developed, these simplified distinctions have largely fallen out of favour in recent years. There is an ongoing and intense debate on the ambiguous conceptualizations of implicit attitudes and implicit measures (see Corneille & Hütter, 2020). For example, Gawronski and Corneille convincingly argue that, despite common claims of unawareness of attitudes, “there is no empirical evidence supporting the idea that implicit measures […] would capture attitudes that people are unaware of” (Gawronski & Corneille, 2025, p. 5; Hahn & Goedderz, 2020). Thus, if SJT theorizing aims to uphold claims regarding unconscious internalization of stigma, empirical support for this claim is needed. Overall, previous assumptions regarding unique or even superior characteristics of implicit measures over explicit measures—such as uncontrollability, low susceptibility to demand effects, self‐presentation concerns, social desirability or faking intentions, and access to automatic processes or unconscious attitudes—have been largely discredited (Corneille & Gawronski, 2024). This does not entirely question the validity of an IAT, which can be interpreted as an indirect indicator of evaluative representations of social groups inferred from behavioural performance indicators. However, we should abandon the idea that these are clear indicators of intentionally hidden or unconscious attitudes. Given psychometric weaknesses of these measures, Corneille and Gawronski (2024) propose to employ self‐report measures that are superior in terms of reliability and can be employed under conditions that favour honest responding of personal attitudes or even prompt participants' responses under conditions favourable to automatic processes (see also Ranganath et al., 2008). For example, in our online survey, which employed a completely anonymous data collection procedure, we find no compelling reason to believe that participants would intentionally conceal their personal attitudes in the self‐report measures.

We hasten to add that even if future research provides more compelling empirical evidence supporting the assumption that group attitudes are activated outside people's conscious awareness, such findings still do not support the inference that these attitudes were *formed* via an unconscious internalization or justification process. There is ample research documenting that automatically activated attitudes can arise from deliberative, propositional thinking (e.g. Gawronski et al., 2017). Thus, empirical proof validating SJTs assertion of unconsciously operating system justification processes is still lacking. At the current point in time, we agree with other critiques of SJT (e.g. Owuamalam et al., 2018), in that there is neither sufficient theoretical nor empirical justification for attributing group attitudes among members of disadvantaged groups to unconscious processes of stigma internalization and system justification.

## CONCLUSION

Our research contributes valuable first insights regarding the relationship between group attitudes and ingroup evaluation norms among disadvantaged groups. We acknowledge its limitations in generalizability, which calls for future research with a broader range of disadvantaged groups and diverse attitude and norm measures. Furthermore, examining perceptions of the interplay of positive and negative evaluation norms of different reference groups could provide an even more comprehensive picture. Additionally, we recommend extending the current cross‐sectional correlational approach by incorporating longitudinal and experimental research methodologies. We also believe that integration with research on third variables affecting both the group's relative social status or stigma as well as group attitudes and ingroup evaluation norms, including stigma characteristics (Pachankis et al., 2018), group characteristics such as group permeability or entitativity (Rydell & McConnell, 2005), and individual characteristics such as ideological orientation, identity centrality, and stigma consciousness (Pinel, 1999), might be fruitful in investigating differences in group attitudes and their relationships with social norm evaluations within and between disadvantaged groups. The present research might not only inform the theoretical refinement of SJT but also other models that focus on group attitudes among members of disadvantaged groups, such as the rejection identification model (Branscombe et al., 1999) or the social identity model of system attitudes (Owuamalam et al., 2018). In essence, we emphasize the need for refined theoretical approaches to group attitudes in disadvantaged groups, acknowledging the heterogeneity in group evaluations and norm expressions, and addressing the conceptual ambiguities of internalized inferiority and the use of indirect and direct methods.

## AUTHOR CONTRIBUTIONS

**Juliane Degner:** Conceptualization; investigation; funding acquisition; writing – original draft; methodology; writing – review and editing; formal analysis. **Joelle‐Cathrin Flöther:** Conceptualization; writing – review and editing. **Iniobong Essien:** Conceptualization; writing – review and editing.

## CONFLICT OF INTEREST STATEMENT

None to declare.

## Supporting information


Appendix S1

## Data Availability

The data that support the findings of this study are available in the Open Science Foundation repository (https://osf.io/jrt7v/).

## Footnotes

^1^
Note that SIMSA similarly posits that, in certain contexts, conformity to ingroup norms interacts with group attitudes, particularly among individuals with a strong ingroup identification. However, their Hypothesis 8 focuses on ingroup norms that prescribe ingroup *negativity* (e.g., Rubin et al., 2023). Thus, according to SIMSA, individuals belonging to low‐status groups are expected to exhibit ingroup negativity and/or outgroup preferences when they identify highly with the ingroup and when ingroup norms advocate for system justifying group attitudes.

^2^
The BMI is an imperfect way to estimate individual weight status (American Medical Association, 2023). We nevertheless used it as screening criterion because Prolific did not include any other information related to weight status.

^3^
While the general approach of group‐level and individual level analyses for our hypotheses test calls for applying a multilevel‐analyses approach, the current low number of 3 groups does not provide sufficient power on the group level to run these analyses.
