# Supplementary material for: Pressured to be proud? Investigating the link between perceived norms and intergroup attitudes in members of disadvantaged minority groups
Source: Br J Soc Psychol. 2025 Mar 4;64(2):e12874. doi: 10.1111/bjso.12874 (PMC11877989; doi:10.1111/bjso.12874)
Supplement: Supplementary file 1 — Appendix S1 [file BJSO-64-0-s001.pdf]

Pressured to be Proud?
Investigating the Link between Perceived Norms and Intergroup Attitudes in Members of Disadvantaged Minority Groups

Juliane Degner[1] | **Joelle-Cathrin Flöther**[1] | **Iniobong Essien**[2]

SUPPLEMENT

[1]University of Hamburg, Germany

[2]Leuphana Universität Lüneburg, Germany

Correspondence
Juliane Degner, Department of Social Psychology, Universität Hamburg, Von-Melle-Park 5, Hamburg 20146, Germany.
Email: juliane.degner@uni-hamburg.de

Funding information
Excellence Strategy of the Federal Ministry of Education and Research (BMBF) of Germany and the Free and Hanseatic City of Hamburg

Acknowledgments
We thank Leonie Asche, Pauline Ellerman, and Linda Sinn for their valuable help with data collection.

Table of Contents

S.1 Extended Analyses of Interrelations between group evaluations and evaluative norm perceptions.

S.2 Multiple regressions testing for independent predictive value of different norms.

S.3 Additional Measures.
- S.3.1 Materials
- S.3.2 Descriptive Statistics
- S.3.3 Exploratory analysis: Does ingroup identity centrality moderate the relationships between ingroup norms and group evaluations?
- S.3.4 Exploratory analysis: Do system justifying beliefs moderate the relationship between societal norms and group evaluations?
- S.3.5 Exploratory analysis: Does political ideology moderate the relationship between ingroup norms and group evaluations?

© 2025 The Author(s).

S.1. Extended Analyses of Interrelations between group evaluations and evaluative norm perceptions.

For hypotheses tests of interrelations between group evaluations and evaluation norms, we present the results of a series of hierarchical multiple regression analyses for each group evaluation measure, respectively. In these regressions, we first entered the mean-centered norm perception measure as predictor in order to test for a general relationship between evaluation and norm measures. In the second step, we entered two dummy variables coding group membership (with Sample 1 [Gay and Lesbian participants] as reference) in order to test whether relationships between evaluation and norm measures were confounded with mean group differences in both variables – that's the relevant group-level effect. In the third step we entered the interaction terms of norm and dummy variables to test for moderation of the relationship by type of group.

S.1.1 IAT scores.

The first hierarchical multiple regression used the perceived descriptive ingroup norm as (centered) predictor of the IAT score (see Table 1). At step 1, it revealed a significant and strong relationship between IAT score and perceived descriptive ingroup norm, ß =.392, $t(589) = 10.436$, $p < .001$. Entering the dummy variables coding samples in step 2 considerably diminished this relationship, ß =.160, $t(589) =$ 2.323, $p = .021$. None of the interaction terms in Step 3 were significant, indicating that the relationship between IAT score and perceived descriptive norm was not moderated by sample. As can be seen in Figure 1 (Panel A), the relationship between IAT scores and perceptions of descriptive norms were mainly based on group differences in norms with very low within-group variance relating to the IAT.

The second hierarchical multiple regression used the perceived injunctive ingroup norm as (centered) predictor of the IAT score. At step 1, it revealed a significant relationship between IAT score and perceived injunctive ingroup norm, ß =.372, $t(589) = 9.727$, $p < .001$. Entering the dummy variables coding samples in step 2 rendered this relationship non-significant, ß =.105, $t(587) = 1.515$, $p = .130$, indicating that it was by and largely driven by sample differences in mean scores. Entering the interaction terms in Step 3 again revealed no significant moderation effects by group (Panel B Figure 1).

The third hierarchical multiple regression used the perceived injunctive societal norm as (centered) predictor of the IAT score. Again, it revealed a significant relationship between IAT score and perceived injunctive societal norm, ß =.169, $t(589) = 4.160$, $p < .001$ at Step 1. Entering the dummy variables coding samples in step 2 considerably diminished this relationship, ß = -.073, $t(587) = 1.665$, $p = .096$. Again, none of the interaction terms in Step 3 were significant, indicating that the relationship between IAT score and perceived descriptive norm was not moderated by sample (panel C Figure 1).

S.1.2 Intergroup Preference Item.

Again, we conducted multiple regression analyses using the three different norm measures as predictors of the one-item group preference score. The first hierarchical multiple regression used the perceived descriptive ingroup norm as (centered) predictor. At step1, it revealed a significant relationship between group preference and perceived descriptive ingroup norm, ß =.474, $t(604) = 13.231$, $p < .001$. Entering the dummy variables coding samples in step 2 considerably diminished this relationship, but it remained significant, ß = .200, $t(602) = 3.023$, $p < .001$. Entering the interaction terms in Step 3 revealed no effect for the contrast between samples 1 and 2 (gay vs. black), ß = -.020, $t(600) = 0.267$, $p = .790$, but a marginally significant moderation effect of the contrast between sample 2 and 3, ß = -.200, $t(600) = 1.929$, $p = .054$. As can be seen in Table 3 and Figure 1 in the main manuscript, there were small positive correlations in the gay sample and the black sample, indicating that ingroup preference was positively related to perceptions of a descriptive norm of ingroup positivity, but no significant correlation was observed in the weight sample.

The second hierarchical multiple regression used the perceived injunctive ingroup norm as (centered) predictor of the one-item group preference measure. At step 1, it revealed a significant relationship between group preference and perceived injunctive ingroup norm, ß =.412, $t(604) = 11.113$, $p < .001$.

Entering the dummy variables coding samples in step 2 rendered this relationship non-significant, ß =-.018, $t(602) = -0.272$, $p = .786$, indicating that it was entirely driven by sample differences in mean scores of both variables. Entering the interaction terms in in Step 3 revealed no effect for the contrast between samples 1 and 2 (gay vs. black), ß = -.037, $t(600) = 0.442$, $p = .659$, but a marginally significant moderation effect of the contrast between sample 2 and 3, ß = -.181, $t(600) = 1.677$, $p = .094$. Thus, whereas there were no significant correlations within the gay sample and the Black sample, there was a significant negative correlation in the overweight sample indicating that participants tended to express more outgroup preference the more they perceived a injunctive ingroup norm towards positive ingroup evaluation.

The third hierarchical multiple regression used the perceived injunctive societal norm as (centered) predictor of the one-item group preference measure. At step 1, it revealed a significant relationship between group preference and perceived injunctive societal norm, ß =.166, $t(604) = 4.134$, $p < .001$. Entering the dummy variables coding samples in step 2 lead to a reversed effect, ß = -.153, $t(602) = -3.652$, $p < .001$. Both interaction terms entered in Step 3 were significant, ß = .148, $t(600) = 2.414$, $p = .016$ and ß = .127, $t(600) = 2.020$, $p = .044$, indicating significantly different relationships in all three samples. As can be seen in Table 3 and Figure 1 in the main manuscript, within-group variance in self-reported group preference was always negatively related to perceived societal norms, whereas there was a positive relationship between these variables on the group level.

S.1.3 Ingroup Evaluation Scale.

Finally, we also conducted multiple regression analyses using the three different norm measures as predictors of the positive ingroup evaluation scale. The first hierarchical multiple regression used the perceived descriptive ingroup norm as (centered) predictor. At step 1, it revealed a large significant relationship between positive ingroup evaluation and perceived descriptive ingroup norm, ß =.867, $t(604) = 42.840$, $p < .001$. Entering the dummy variables coding samples in step 2 considerably diminished this relationship, but it remained significant, ß = .463, $t(602) = 13.926$, $p < .001$. Entering the interaction terms in Step 3 revealed no significant moderation by group membership. As can be seen in Table 3 and Figure 1 in the main manuscript, there were substantial positive correlations between both variables in all three samples, indicating that own ingroup evaluations were strongly related to perceptions of a descriptive ingroup norm of group evaluation on both the individual and the group level.

The second hierarchical multiple regression used the perceived injunctive ingroup norm as (centered) predictor of positive ingroup evaluations and yielded very similar results. At step 1, we observed a significant relationship between group evaluation and perceived injunctive ingroup norm, ß =.814, $t(604) = 34.491$, $p < .001$. Again, entering the dummy variables coding the three samples in step 2 strongly diminished this relationship, ß = .272, $t(602) = 7.417$, $p < .001$, indicating that it was partly driven by sample differences in mean scores of both variables. Entering the interaction terms in in Step 3 revealed a marginal moderation effect for the contrast between samples 1 and 2 (gay vs. black), ß = .085, $t(600) = 1.866$, $p = .063$, and a significant moderation effect of the contrast between sample 2 and 3, ß = .138, $t(600) = 2.329$, $p = .020$. Bivariate correlations calculated separately for each sample revealed a non-significant positive correlation in the gay sample, a larger and significant correlation in the Black sample and an even larger significant positive correlation in the overweight sample (see Table 3 in the main manuscript). Thus, in all samples, perceived descriptive ingroup norm and own group evaluation were interrelated, but most strongly so in the overweight sample.

The third hierarchical multiple regression used the perceived injunctive societal norm as (centered) predictor of positive ingroup evaluations. At step 1, it revealed a significant relationship between ingroup evaluation and perceived injunctive societal norm, ß =.501, $t(604) = 14.221$, $p < .001$. Entering the dummy variables coding samples in step 2 eliminated this effect, ß = .016, $t(602) = 0.679$, $p = .497$, indicating that this relationship was entirely driven by group level differences. Of the two interaction terms entered in Step 3, the first contrast was not significant, ß = .038, $t(600) = 1.070$, $p = .285$, but the contrast race vs. overweight was, ß = .146, $t(600) = 4.041$, $p < .001$, indicating a significant moderation. Zero-order correlations listed in Table 3 in the main manuscript indicate a non-significant negative relationship in the gay sample, a null-effect in the black sample and significant positive relationship in the overweight sample (see also Figure 1 in the main manuscript).

Table S.1. Summary results of the hierarchical regression analyses (unstandardized coefficients and their SEs)

	IAT			Group preference item			Group evaluation scale		
	Ingroup descriptive	Ingroup injunctive	Society injunctive	Ingroup descriptive	Ingroup injunctive	Society injunctive	Ingroup descriptive	Ingroup injunctive	Society injunctive
**Step1 R^2**	**.154**	**.138**	**.029**	**.225**	**.170**	**.028**	**.752**	**.663**	**.251**
constant	-0.066 (.020)	-0.067 (.020)	-0.067 (.021)	4.662 (.048)	4.662 (.050)	4.662 (.054)	4.534 (.042)	4.534 (.049)	4.534 (.073)
Norm	0.117 (.011)	0.114 (.012)	0.061 (.015)	0.373 (.028)	0.334 (.030)	0.157 (.038)	1.043 (.024)	1.007 (.029)	0.721 (.051)
**Step 2 R^2 / ΔR^2**	**.256 / .103**	**.253 / .114**	**.253 / .225**	**.287 / .062**	**.276 / .106**	**.292 / .264**	**.820 / .068**	**.781 / .119**	**.762 / .512**
constant	0.209 (.036)	0.223 (.036)	0.256 (.033)	5.238 (.092)	5.378 (.092)	5.416 (.083)	5.312 (.071)	5.513 (.077)	5.759 (.073)
Norm	0.048 (.021)	0.032 (.021)	-0.026 (.016)	0.158 (.052)	-0.015 (.054)	-0.144 (.039)	0.556 (.040)	0.336 (.045)	0.024 (.102)
Dummy1	-0.321 (.046)	-0.317 (.047)	-0.287 (.046)	-0.479 (.117)	-0.399 (.119)	-0.339 (.116)	-0.120 (.090)	-0.040 (.100)	0.122 (.102)
Dummy2	-0.492 (.044)	-0.540 (.073)	-0.667 (.050)	-1.215 (.186)	-1.700 (.115)	-1.869 (.127)	-2.148 (.143)	-2.811 (.156)	-3.687 (.112)
**Step 3 R^2 / ΔR^2**	**.257 /.000**	**.256 / .004**	**.254 / .001**	**.292 / .005**	**.283 / .007**	**.299 / .008**	**.821 / .001**	**.783 / .002**	**.768 / .007**
constant	0.197 (.044)	0.182 (.045)	0.251 (.034)	5.154 (.095)	5.310 (.115)	5.470 (.085)	5.347 (.073)	5.653 (.096)	5.807 (.074)
Norm	0.062 (.037)	0.086 (.042)	-0.010 (.029)	0.260 (.095)	0.076 (.107)	-0.305 (.074)	0.513 (.073)	0.151 (.089)	-0.117 (.065)
Dummy1	-0.298 (.071)	-0.284 (.078)	-0.275 (.050)	-0.481 (.181)	-0.531 (.196)	-0.448 (.126)	-0.105 (.139)	-0.245 (.164)	0.116 (.110)
Dummy2	-0.488 (.088)	-0.561 (.081)	-0.664 (.058)	-1.418 (.220)	-1.885 (.206)	-1.863 (.143)	-2.042 (.169)	-2.813 (.172)	-3.475 (.125)
IA1	-0.024 (.052)	-0.047 (.059)	-0.025 (.038)	-0.035 (.131)	0.067 (.151)	0.228 (.095)	0.003 (.101)	0.236 (.126)	0.089 (.083)
IA2	-0.018 (.050)	-0.086 (.052)	-0.018 (.043)	-0.246 (.127)	-0.221 (.132)	0.215 (.106)	0.114 (.098)	0.257 (.110)	0.376 (.093)

** p <.01, * p <.05; Dummy1 = contrast gay vs. black, Dummy2 = contrast black vs. overweight,
IA1 = interaction Dummy 1 x norm measure, IA2 = interaction Dummy2 x norm measure

S.2 Multiple regressions testing for independent predictive value of different norms.

For each group evaluation measure, we calculated additional multiple regression analyses simultaneously including all three norm measures as predictors of each group evaluation measure to explore for independent effects of these norms.

S.2.1 IAT scores.

To test for the independent versus interactive effects of ingroup and societal norms, we conducted a multiple regression analysis including all three norm measures as simultaneous predictors of D-scores, $R^2 = .165$, $F(3, 587) = 38.767$, $p < .001$. We observed independent predictive values of all norm measures with ß =.325, $t(587) = 3.569$, $p < .001$ for the descriptive ingroup norm, ß =.163, $t(587) = 1.715$, $p = .087$ for the prescriptive ingroup norm, and ß =-.133, $t(587) = -2.682$, $p = .008$ for the prescriptive societal norm. Inclusion of the dummy variables controlling for sample, $R^2 = .264$, $F(5, 585) = 41.954$, $p < .001$, substantially reduced the predictive value of the descriptive ingroup norm, ß =.169, $t(585) = 1.847$, $p = .065$, and the prescriptive ingroup norm, ß =.056, $t(585) = 0.590$, $p = .555$, but not the prescriptive societal norm, ß =-.114, $t(585) = -2.436$, $p = .015$. The significant beta coefficients of both dummy variables, ß =-.284, $t(585) = -6.673$, $p < .001$, and ß = -.459, $t(585) = -6.396$, $p < .001$, indicate strong effects of group differences in all three norm variables.

S.2.2 Intergroup Preference Item.

We investigated the independent effects of each norm measure on self-reported intergroup preferences by conducting a multiple regression analysis including all three norm measures as simultaneous predictors of self-reported group preferences, $R^2 = .246$, $F(3, 602) = 65.519$, $p < .001$. We observed independent predictive values of the descriptive group norm, ß =.593, $t(602) = 6.953$, $p < .001$, and of the prescriptive societal norm, ß = -.181, $t(602) = -3.927$, $p < .001$, but no independent effect of prescriptive ingroup norms, ß =-.012, $t(602) = -0.138$, $p = .890$. Inclusion of the dummy variables coding groups, $R^2 = .317$, $F(5, 600) = 55.574$, $p < .001$, revealed significant group differences for both dummy variables, ß = -.137, $t(600) = -3.384$, $p < .001$, and ß = -.500, $t(600) = -7.376$, $p < .001$, and independent predictive effect for all three norm variables, with ß =.394, $t(600) = 4.525$, $p < .001$, for the descriptive ingroup norm, ß = -.182, $t(600) = -2.024$, $p = .043$, for the prescriptive ingroup norm and ß = -.177, $t(600) = -4.025$, $p < .001$, for the prescriptive societal norm.

S.2.3 Ingroup Evaluation Scale.

Again, we investigated the independent effects of ingroup norms and societal norms on ingroup evaluations with a multiple regression analysis including all three norm measures as simultaneous predictors of the open expression of positive ingroup evaluations, $R^2 = .758$, $F(3, 602) = 627.803$, $p < .001$. We observed independent predictive values of the descriptive group norm, ß =.739, $t(602) = 15.281$, $p < .100$, and the prescriptive ingroup norm, ß = .176, $t(602) = 3.499$, $p < .100$, and a small negative independent effect of prescriptive societal norms, ß = -.053, $t(602) = -2.033$, $p = .042$. Inclusion of the dummy variables, $R^2 = .822$, $F(5, 600) = 557.947$, $p < .001$, revealed no significant group differences between the Gay and Lesbian sample and the Black and African American sample, ß = -.019, $t(600) = -0.925$, $p = .355$, but a significant difference for the higher weight sample, ß = -.060, $t(600) = -2.860$, $p < .001$.

S.3 Additional Measures.

S.3.1 Materials

Status Perceptions. Participants completed a two-item adaption of the MacArthur scale to measure perceived status differences of social groups in society. We presented a ladder with 10 rungs, ranging from 1 (*lowest status*) to 10 (*highest status*) along with one item asking participants where they thought [Gay / Black / Overweight people] in the US stand on this ladder in general and where they thought [Straight / White / normal-weight people] in the US stand on this ladder in general.

Ingroup Centrality. We employed two items adapted from Leach and colleagues (2008) to measure the centrality of ingroup membership to one's identity (i.e., "*Being Gay / Black / Overweight is an important part of my identity.", "Being Gay / Black / Overweight is an important part of how I see myself."*).

Ideology. For exploratory reasons, we included the eight-item System Justification Scale (Jost & Thomson, 2000) measuring participants' perceptions of the fairness, legitimacy, and justifiability of the prevailing social system. In addition, we asked participants to indicate their general political views and ideology on a single item with a 7-point scale ranging from *extremely liberal* to *extremely conservative.*

S.3.2 Descriptive Statistics

Samples were characterized by high levels of identity centrality and low levels of system justifying beliefs and skewed fairly liberal (see Table S.2.1 and S.2.2). There were significant group differences with regard to ingroup centrality, $F(2,603) = 253.999, p < .001, \eta^2_p = .457$. As can be seen in Table 2, Gay and Lesbian participants as well as Black and African American participants exhibited very high levels of ingroup centrality, whereas Overweight participants tended to reject ingroup centrality items. We also observed small group differences with regard to system justifying beliefs, $F(2,603) = 3.660, p = .045, \eta^2_p = .010$, but all three groups exhibited very low mean levels, indicating a general tendency to reject system justifying statements. Similarly, there were significant group differences in ideology, $F(2,603) = 17.702, p < .001, \eta^2_p = .055$, with Gay and Lesbian participants reporting to be more progressive than Black and Overweight participants.

One may thus assume, that the relationship between individual group attitudes and societal norm compliance is moderated by individual strength of the system justification motive. Alternatively, one could also argue that societal pressure leads to mere instrumental public conformity (going along to get along), whereas the more self-relevant ingroup norms trigger private conformity, potentially moderated by the degree of ingroup identification.

Table S.3.1. **Group Status Perceptions**

	Ingroup status		Outgroup status		
	M	(*SD*)	*M*	(SD)	*d*
Gay/Lesbian	4.592	(1.311)	7.607	(1.832)	1.349
Black/African American	3.960	(1.542)	8.203	(1.802)	1.652
Overweight	4.139	(1.419)	6.495	(1.513)	1.279

Notes. Cohen's d from paired *t*-test comparing ingroup and outgroup status.

Table S.3.2. **Identity Centrality and System Justification Beliefs**

	Identity centrality			System justification			Ideology		
	M	(*SD*)	*d*	*M*	(*SD*)	*d*	*M*	(*SD*)	*d*
Gay/Lesbian	5.436	(1.451)	0.990	2.510	(1.043)	-1.429	2.168	(2.286)	-1.570
Black/African American	5.943	(1.423)	1.365	2.766	(1.150)	-1.073	2.901	(2.511)	-0.785
Overweight	2.834	(1.613)	-0.723	2.715	(1.051)	-1.222	2.875	(2.555)	-0.716

Notes. Cohen's d from *t*-test against the midpoint of the response scales (4)

Table S.3.3 **Zero-order correlation coefficients *r* (*p*-values) for the relation between group attitude measures and identity centrality, system justification beliefs, and ideology – with partial correlations controlling for sample.**

	Evaluative norm perceptions		
**IAT scores**	**Identity centrality**	**System Justification beliefs**	**Ideology**
Overall (bivariate)	.373 (.000)	-.105 (.011)	-.202 (.000)
Overall (partial)	.147 (.000)	-.079 (.254)	-.121 (.003)
Gay / Lesbian	.184 (.011)	-.171 (.018)	-.240 (.001)
Black / African American	.161 (.024)	.015 (.830)	-.055 (.443)
Higher Weight	.140 (.045)	-.084 (.231)	-.096 (.171)
**Intergroup preference**			
Overall (bivariate)	.523 (.000)	-.183 (.000)	-.218 (.000)
Overall (partial)	.345 (.000)	-.157 (.000)	-.126 (.002)
Gay / Lesbian	.437 (.000)	-.347 (.000)	-.312 (.000)
Black / African American	.322 (.000)	-.134 (.058)	-.187 (.008)
Higher Weight	.170 (.014)	-.059 (.401)	-.021 (.768)
**Ingroup evaluation**			
Overall (bivariate)	.745 (.000)	-.027 (.510)	-.134 (.001)
Overall (partial)	.617 (.000)	.043 (.294)	.018 (.668)
Gay / Lesbian	.529 (.000)	-.142 (.047)	-.214 (.003)
Black / African American	.655 (.000)	.082 (.247)	-.134 (.058)
Higher Weight	.203 (.003)	.046 (.510)	.032 (.642)

S.3.4 Exploratory partial correlations:
Are group-level correlations between group attitudes and norm perceptions mediated by social identity or ideological variables?

	Evaluative norm perceptions		
**IAT scores**	**Descriptive Ingroup Norm**	**Prescriptive Ingroup Norm**	**Prescriptive Societal Norm**
Overall bivariate correlation	.392 (.000)	.372 (.000)	.169 (.000)
Controlling for Identity Centrality	.211 (.000)	.199 (.000)	.052 (.206)
Controlling for System Justification beliefs	.400 (.000)	.378 (.000)	.205 (.000)
Controlling for Ideology	.385 (.000)	.368 (.000)	.186 (.000)
**Intergroup preference**			
Overall bivariate correlation	.474 (.000)	.412 (.000)	.166 (.000)
Controlling for Identity Centrality	.212 (.000)	.150 (.000)	.007 (.874)
Controlling for System Justification beliefs	.494 (.000)	.429 (.000)	.234 (.000)
Controlling for Ideology	.472 (.000)	.413 (.000)	.196 (.000)
**Ingroup evaluation**			
Overall bivariate correlation	.867 (.000)	.814 (.000)	.501 (.000)
Controlling for Identity Centrality	.753 (.000)	.689 (.000)	.415 (.000)
Controlling for System Justification beliefs	.870 (.000)	.817 (.000)	.533 (.000)
Controlling for Ideology	.867 (.000)	.816 (.000)	.522 (.000)

S.3.3 Exploratory analysis:

Does ingroup identity centrality moderate the relationships between ingroup norms and group evaluations?

We explored whether the relationships between the three group evaluation measures and perceptions of descriptive ingroup norms and injunctive ingroup norms were moderated by participants' levels of ingroup identification, as measure with the ingroup centrality subscale.

We thus conducted a series of hierarchical regression analyses, including identity centrality and the product of identity centrality and the respective norm measures. Whenever we observed a significant moderation effect, we split the sample into high, moderate, and low identifiers for further exploratory inspection of the pattern of results.

Descriptive ingroup norms and IAT scores.

The multiple regression analyses using IAT scores as criterion documented a significant positive effect of descriptive ingroup norms, ß = .259, $t(588) = 5.237$, $p < .001$, and a simultaneous significant positive effect of identity centrality, ß = .205, $t(588) = 4.145$, $p < .001$, indicating that participants tended to exhibit higher ingroup preference in the IAT, the more they perceived a descriptive ingroup norm towards ingroup positivity and the more they viewed their group membership as central to their identity. We did not observe a significant moderation effect, ß = -.165, $t(587) = 0.929$, $p = .353$.

Descriptive ingroup norms and intergroup preferences.

The multiple regression analyses using self-reported intergroup preferences as criterion documented a significant positive effect of descriptive ingroup norms, ß = .232, $t(603) = 5.161$, $p < .001$, and a simultaneous significant positive effect of identity centrality, ß = .371, $t(603) = 8.276$, $p < .001$, indicating that participants expressed higher ingroup preferences, the more they perceived a descriptive ingroup norm towards ingroup positivity and the more they viewed their group membership as central to their identity. We did observe a marginally significant moderation effect, ß = .321, $t(602) = 1.995$ $p = .046$. Further exploration indicated that the relationship between group preference and descriptive ingroup norms was somewhat stronger among participants with low identity centrality, $r(188) = .144$, $p = .048$, substantially higher in participants with moderate identity centrality, $r(245) = .385$, $p < .001$, but nonsignificant in participants with highest identity centrality, $r(173) = .066$, $p = .390$. Note, however, that the latter group has severely restricted variance in both the group preference as well as the norm perception measure, thus limiting the possibility of observing significant correlations.

Injunctive ingroup norms and IAT scores.

The multiple regression analyses using IAT scores as criterion documented a significant positive effect of injunctive ingroup norms, ß = .231, $t(588) = 4.931$, $p < .001$, and a simultaneous significant positive effect of identity centrality, ß = .235, $t(588) = 5.007$, $p < .001$, indicating that participants exhibited higher ingroup preference in the IAT, the more they perceived a injunctive ingroup norm towards ingroup positivity and the more they viewed their group membership as central to their identity. We did not observe a significant moderation effect, ß = -.127, $t(587) = 0.696$, $p = .487$.

Injunctive ingroup norms and intergroup preferences.

The multiple regression analyses using self-reported intergroup preferences as criterion documented a significant positive effect of descriptive ingroup norms, ß = .154, $t(603) = 3.578$, $p < .001$, and a simultaneous significant positive effect of identity centrality, ß = .430, $t(603) = 10.014$, $p < .001$, indicating that participants expressed higher ingroup preferences, the more they perceived a descriptive ingroup norm towards ingroup positivity and the more they viewed their group membership as central to their identity. We did observe a marginally significant moderation effect, ß = .411, $t(602) = 2.474$ $p = .014$. Further exploration indicated that the relationship between group preference and injunctive ingroup norms was nonsignificant in participants with very low ingroup centrality, $r(188) = .034$, $p = .639$, robust and of moderate effect size among participants with moderate identity centrality, $r(245) = .366$, $p < .001$, and again non-significant in participants with high identity centrality, $r(173) = -.014$, $p = .856$.

Injunctive ingroup norms and ingroup evaluations.

The multiple regression analyses using self-reported ingroup evaluations as criterion documented a significant positive effect of injunctive ingroup norms, ß = .574, $t(603) = 23.293$, $p < .001$, and a simultaneous significant positive effect of identity centrality, ß = .401, $t(603) = 16.278$, $p < .001$, indicating that participants expressed higher ingroup preferences, the more they perceived an injunctive ingroup norm towards ingroup positivity and the more they viewed their group membership as central to their identity. The moderation effect missed the significance criterion, ß = .181, $t(602) = 1.898$ $p = .058$. Further exploration indicated that the relationship between group preference and injunctive ingroup norms was substantially bigger in participants with low ingroup centrality, $r(188) = .690$, $p < .001$ and with moderate identity centrality, $r(245) = .781$, $p < .001$, compared to a smaller effect in participants with high identity centrality, $r(173) = .456$, $p < .001$.

To summarize, the relationships between group evaluation measures and participants perceptions of descriptive and injunctive ingroup norms were not systematically moderated by ingroup centrality. In cases where identity centrality mattered, we observed the closest relationship between ingroup norm perceptions and group evaluations for those participants with moderated ingroup centrality. While superficially, these results appear to contradict SIT assumptions that ingroup norms should matter most for those who identify highly with the group. However, given that this group is also the one with the lowest variance in their perception of ingroup norms, it is little surprising that no statistically significant correlation can be observed: These group members largely agree in their perception of ingroup norms and in their group evaluations – thus actually aligning their group attitudes extremely closely with descriptive ingroup norms.

S.3.4 Exploratory analysis:

Do system justifying beliefs moderate the relationship between societal norms and group evaluations?

We investigated whether the relationship between the three group evaluation measures and perceptions of injunctive societal norms was moderated by participants level of system justification beliefs. Note that results of these analyses are highly restricted in generalizability because our samples in general exhibited very low levels of system justifying beliefs, in general rejecting the notion that society was fair.

Injunctive Societal norms and IAT scores.

The multiple regression analyses using IAT scores as criterion documented a significant positive effect of injunctive societal norms, ß = .211, $t(588) = 5.074$, $p < .001$, and a simultaneous significant negative effect of system justifying believes, ß = -.160, $t(588) = -3.856$, $p < .001$, indicating that participants tended to exhibit higher ingroup preference the more they perceived a societal norm towards ingroup positivity and the more they rejected system justifying statements. We did not observe a significant moderation effect by system justifying believes though, ß = .097, $t(587) = 0.634$, $p = .526$.

Injunctive Societal norms and intergroup preferences.

The multiple regression analyses using self-reported intergroup preference as criterion similarly documented a significant positive effect of injunctive societal norms, ß = .235, $t(603) = 5.785$, $p < .001$, and a simultaneous significant negative effect of system justifying believes, ß = -.248, $t(603) = -6.116$, $p < .001$, indicating that participants tended to exhibit higher ingroup preference the more they perceived a societal norm towards ingroup positivity and the more they rejected system justifying statements. We did not observe a significant moderation effect by system justifying believes though, ß = .017, $t(602) = 0.110$, $p = .913$.

Injunctive Societal norms and ingroup evaluations.

Finally, the multiple regression analyses using self-reported positive ingroup evaluations as criterion documented a significant positive effect of injunctive societal norms, ß = .550, $t(603) = 15.313$, $p < .001$, and a simultaneous significant negative effect of system justifying believes, ß = -.179, $t(603) = -4.987$, $p < .001$, indicating that participants tended to exhibit higher ingroup preference the more they perceived a societal norm towards ingroup positivity and the more they rejected system justifying statements. We did observed a marginally significant moderation effect by system justifying believes, ß = .260, $t(602) = 1.949$, $p = .052$. Further exploratory analyses indicated that minority group members with the lowest SJT scores,

thus those who rejected system justification beliefs the most, exhibited lower correlations between ingroup evaluation and societal norms $r(203) = .423, p < .011$, than for those with moderate rejection, $r(208) = .561, p < .011$ and those with lowest rejection $r(195) = .602, p < .011$. In summary, we observed some support that rejection of system justifying beliefs reduced the relationship between group evaluations and perceived societal norms. However, given the very low levels of system justifying beliefs in our samples, the starting notion that participants exhibit a strong motivation to legitimize and support the existing societal system that disadvantages their own ingroup should be questioned.

S.3.5 Exploratory analysis:

Does political ideology moderate the relationship between ingroup norms and group evaluations?

We investigated whether the relationship between the three group evaluation measures and perceptions of ingroup norms was moderated by participants ideological orientations. Note that results of these analyses are highly restricted in generalizability because our samples exhibited a strong progressive-leaning tendency with rather restricted variance in political ideology.

Descriptive ingroup norms and IAT scores.

The multiple regression analysis using IAT scores as criterion documented a significant positive effect of descriptive ingroup norms, ß = .378, $t(587) = 10.117, p < .001$, and a simultaneous significant negative effect of political ideology, ß = -.171, $t(587) = -4.586, p < .001$, indicating that participants tended to exhibit higher ingroup preference the more they perceived a descriptive ingroup norm towards ingroup positivity and the more they described themselves as progressive. We did not observe a non-significant moderation effect by political ideology, ß = -.199, $t(587) = -1.834, p = .067$.

Descriptive ingroup norm and intergroup preferences.

The multiple regression analyses using self-reported intergroup preference as criterion similarly documented a significant positive effect of descriptive ingroup norms, ß = .460, $t(602) = 13.088$, $p < .001$, and a simultaneous significant negative effect of political ideology, ß = -.183, $t(603) = -5.211, p < .001$, indicating that participants tended to exhibit higher ingroup preference the more they perceived a descriptive ingroup norm towards ingroup positivity and the more progressive they describe themselves. We did also observe a significant moderation effect by political ideology, ß = -.280, $t(602) = -2.723, p = .007$. Further exploratory analyses indicated that minority group members with the strongest progressive ideology exhibited higher correlations between self-reported ingroup preference and descriptive ingroup norms $r(144) = .612, p < .011$, than for those with moderate progressiveness, $r(295) = .446, p < .001$ and those with xx $r(167) = .391, p < .011$.

Descriptive ingroup norms and ingroup evaluations.

The multiple regression analysis using self-reported positive ingroup evaluations as criterion documented a significant positive effect of descriptive ingroup norms, ß = .867, $t(602) = 42.480$, $p < .001$, and a simultaneous significant negative effect of political ideology, ß = -.069, $t(602) = -3.445, p = .001$, indicating that participants tended to exhibit higher ingroup positivity the more they perceived a descriptive ingroup norm towards ingroup positivity and the more they described themselves as progressive. We did not observe a significant moderation effect by political ideology, ß = -.048, $t(602) = -0.810, p = .418$.

Injunctive ingroup norms and IAT scores.

The multiple regression analysis using IAT scores as criterion documented a significant positive effect of injunctive ingroup norms, ß = .362, $t(587) = 9.612, p < .001$, and a simultaneous significant negative effect of political ideology, ß = -.181, $t(587) = -4.812, p < .001$, indicating that participants tended to exhibit higher ingroup preference the more they perceived an injunctive ingroup norm towards ingroup positivity and the more they described themselves as progressive. We did not observe a significant moderation effect by political ideology, ß = -.192, $t(587) = -1.623, p = .105$.

Injunctive ingroup norm and intergroup preferences.

The multiple regression analyses using self-reported intergroup preference as criterion similarly documented a significant positive effect of descriptive ingroup norms, ß = .401, $t(602) = 11.044$, $p < .001$, and a simultaneous significant negative effect of political ideology, ß = -.195, $t(603) = -5.369, p < .001$, indicating that participants tended to exhibit higher ingroup preference the more they perceived an injunctive ingroup

norm towards ingroup positivity and the more progressive they describe themselves. We did also observe a significant moderation effect by political ideology, ß = -.375, $t(602) = -3.284$, $p = .001$. Further exploratory analyses indicated that minority group members with the strongest progressive ideology exhibited higher correlations between self-reported ingroup preference and injunctive ingroup norms $r(144) = .569$, $p < .001$, than for those with moderate progressiveness, $r(295) = .398$, $p < .001$ and those with lowest progressiveness $r(167) = .293$, $p < .001$.

Injunctive ingroup norms and ingroup evaluations.

The multiple regression analysis using self-reported positive ingroup evaluations as criterion documented a significant positive effect of injunctive ingroup norms, ß = .809, $t(602) = 34.594$, $p < .001$, and a simultaneous significant negative effect of political ideology, ß = -.088, $t(602) = -3.752$, $p < .001$, indicating that participants tended to exhibit higher ingroup positivity the more they perceived an injunctive ingroup norm towards ingroup positivity and the more they described themselves as progressive. We did not observe a significant moderation effect by political ideology, ß = -.102, $t(602) = -1.371$, $p = .171$.

In summary, political ideology seemed to only moderate the relationship between the one-item group preference measure and ingroup norm perceptions. Specifically, those who described themselves as more progressive, exhibited higher alignment between the self-reported group preference and their perception of descriptive and injunctive in group norm. No comparable effects were observed for the IAT or the ingroup evaluation measure, thus questioning the robustness of this finding. Given the restricted variance and progressive-leaning tendencies in our samples, these analyses also have to be interpreted with caution.
